# Strain Virtual Sensing Applied to Industrial Presses for Fatigue Monitoring

**DOI:** 10.3390/s24113354

**Published:** 2024-05-23

**Authors:** Bartomeu Mora, Jon Basurko, Urko Leturiondo, Joseba Albizuri

**Affiliations:** 1 Ikerlan Technology Research Centre, Basque Research and Technology Alliance (BRTA), 20500 Arrasate-Mondragon, Spain; jbasurko@ikerlan.es (J.B.); uleturiondo@ikerlan.es (U.L.); 2 Faculty of Engineering in Bilbao, University of the Basque Country UPV-EHU, 48013 Bilbao, Spain; joseba.albizuri@ehu.eus

**Keywords:** condition monitoring, fatigue, virtual sensing, strain sensor

## Abstract

The techniques that allow one to estimate measurements at the unsensed points of a system are known as virtual sensing. These techniques are useful for the implementation of condition monitoring systems in industrial equipment subjected to high cyclic loads that can cause fatigue damage, such as industrial presses. In this article, three different virtual sensing algorithms for strain estimation are tested using real measurement data obtained from a scaled bed press prototype: two deterministic algorithms (Direct Strain Observer and Least-Squares Strain Estimation) and one stochastic algorithm (Static Strain Kalman Filter). The prototype is subjected to cyclic loads using a hydraulic fatigue testing machine and is sensorized with strain gauges. Results show that sufficiently accurate strain estimations can be obtained using virtual sensing algorithms and a reduced number of strain gauges as input sensors when the monitored structure is subjected to static and quasi-static loads. Results also show that is possible to estimate the initiation of fatigue cracks at critical points of a structural component using virtual strain sensors.

## 1. Introduction

The concept of virtual sensing (VS), also known as soft sensing, refers to the set of methods for obtaining measurements not directly from real sensors but using data inference from other sensors located at other points instead [1].

VS methods can be classified into two main categories: data-driven methods (where a physical model of the system is not used, numerical relations between input and output data being used instead) and model-based methods (where a physical model of the system is used) [2]. Other common classifications of VS methods are deterministic (where uncertainties are not considered) and stochastic (where uncertainties are taken into account) [3,4].

Data-driven VS methods are based on numerical relations between input data and output data (without containing physical parameters that describe the behavior of the system) and require large numbers of samples of real input and output data to be created (known as training data). Commonly used data-driven VS methods are neural networks [5,6] and regression algorithms [7]. Model-based VS methods use physical models of the system, being more complex to implement than data-driven methods and requiring greater knowledge about the physics of the system. Training data is not needed, so these methods are able to represent any situation encompassed by the model physics. The Kalman Filter is a well-known example of a model-based VS method [8]. Some sources refer to data-driven methods as black box methods and model-based methods as white box methods [9].

Deterministic VS methods do not account for uncertainty associated with the input data and the model parameters. The Direct Strain Observer and Least-Squares methods [10] are examples of deterministic model-based methods.

Stochastic VS methods consider that the input data and the parameters of the model have associated uncertainties, using statistical techniques to minimize the error in the provided estimates. Bayesian recursive algorithms, such as the Kalman Filter [8] and its variants (Augmented Kalman Filter for input and states estimation [11], or the Extended Kalman Filter [12] and the Unscented Kalman Filter [13] for non-linear systems) or alternatives such as the Particle Filter [14] are examples of stochastic model-based VS methods.

One of the uses of the VS methods is to provide data for condition monitoring (CM) and structural health monitoring (SHM) systems [15,16]. CM is the process of monitoring industrial machinery in operation and analyzing the obtained data (in real-time or periodically) in order to study the state and health condition of the machine [17]. SHM is similar but applied to structural facilities. Nowadays, CM and SHM systems are often integrated into more complex digital-twin approaches [18].

One of the purposes of CM and SHM systems is fatigue monitoring. The repercussions of fatigue in machines and structures are very important, both from the economic and safety points of view [19]. In industrial machines subjected to large cyclic loads (such as presses and similar machines), fatigue damage is a key issue because the possible breakdowns due to accumulated fatigue can compromise the safety and the economic activity of the equipment.

In the case of CM systems applied to industrial presses and similar machines, vibration measurements obtained with accelerometers are generally used as input data. Some examples can be found in sheet metal-forming presses [20], presses for thermoplastics [21], and paper-production roll presses [22]. To estimate the accumulated fatigue in certain parts of the machine, most fatigue calculation methods use the accumulated cycles of strain/stress [23]; therefore, strain/stress measurement results are of special interest for evaluating the structural health of the monitored machines. In some recent works, strain sensors have been used in presses for calculating the effects of load cycles on critical parts, thereby estimating accumulated fatigue damage. In 2021, an analysis of fatigue crack was performed in a guide bar of a press for automotive components using strain measurements [24]. In 2022, strain sensors were implemented in a metal-forming press to feed a digital twin, with the aim of monitoring the elasto-mechanical behavior of the machine [25]. However, it is not always possible to obtain direct sensor measurements at all locations of interest on a machine, either because there are points where it is not technically feasible to install a sensor (due to harsh environment or lack of accessibility) or because it is of interest to measure a large number of points (which would lead to the installation of too extensive a sensor network). As a solution to these problems, the use of VS technology in CM systems entails both technical and economic advantages.

After reviewing recent developments in VS applied to CM of industrial presses and equivalent machines, it has been concluded that its use is still very sporadic. It has been noticed that there is a lack of consensus on which VS methods are the most suitable for this type of application as well as the appropriate number and position of the input sensors. With the aim of contributing to the absence of information about VS application in industrial presses and similar machines, a scaled prototype of the bed of an industrial press has been designed and built. Using a hydraulic fatigue test machine, load cycles have been applied to the prototype with the objective of causing fatigue damage in the prototype until crack initiation. A Finite Element Method (FEM) model of the prototype has been created to identify the critical areas. The strain and stress in critical areas have been estimated using different VS methods, and with the obtained virtual measurements, the accumulated fatigue in the prototype has been estimated and compared with the results of the real experiment.

Three different model-based VS methods are implemented for strain estimation at the critical areas. Two of them deterministic: the Direct Strain Observer (DSO) and the Least Squares Strain Estimation (LSSE); and the last one stochastic: the Static Strain Kalman Filter (SSKF). These methods have been implemented, and their performance estimating strain at unmeasured points has been compared. The DSO uses external forces measurements as input; meanwhile, the LSSE and the SSKF use only strain measurements as input. Dynamic excitations are not expected in the prototype because the loads applied to the prototype during the fatigue test have a quasi-static nature (as the frequency of the cyclic loads is much lower than the frequency of the first mode of the prototype). Consequently, static models (containing only stiffness parameters) have been used for simplicity instead of a dynamic model (which contains mass, damping, and stiffness parameters) in the implementation of the VS methods.

The overall structure of the paper has been divided into four sections, including this introductory section. Section 2 describes the experimental setup, followed by the description of the methods used in this article. The results and the discussion are presented in Section 3. Finally, the conclusions of the article are presented in Section 4.

## 2. Use Case Description

**Figure 1:**
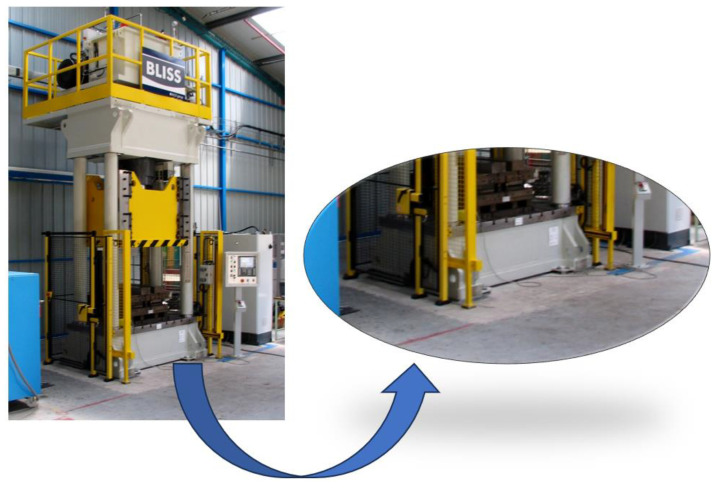
A real industrial press (**left**) and a detailed view of the press bed frame (**right**). Source: https://en.wikipedia.org/wiki/File:Persen.JPG (accessed on 23 May 2024).

The case study consists of the bed of an industrial press, which is the lower part of the complete structure of the machine (see Figure 1), made of welded steel plates. It is a component that must resist significant stress cycles during the operation of the press, so fatigue damage can occur after a long period of use, especially in stress concentration areas such as welded joints.

To carry out the experiment described in this article, a simplified and scaled prototype of the bed of an industrial press has been designed and built. The prototype consists of an assembly manufactured with three steel plates welded together, forming an inverted U-shape. On the inside of the prototype, three reinforcement bars have been welded. The three main plates are welded together with geometric preparation (V-groove type) and full penetration. The reinforcement bars are welded to the main plates (forming T joints) using full penetration fillet welds. The prototype is placed on four simple supports using two fixed steel circular bars.

**Figure 2:**
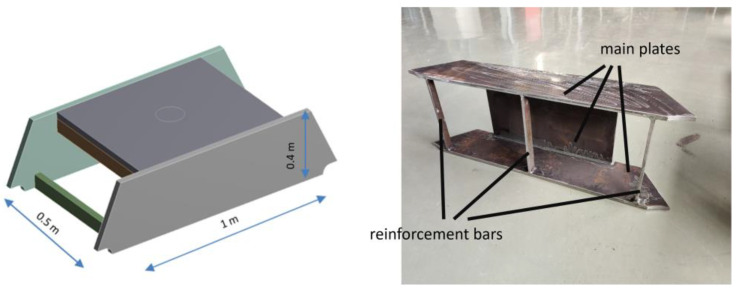
To the **left**, a 3D model of the scaled bed press prototype with the main dimensions included. To the **right**, a real image of the prototype turned on its side.

Table 1 shows the main features of the prototype in terms of weight, size, and materials. Figure 2 shows the simplified prototype used in the paper.

**Figure 3:**
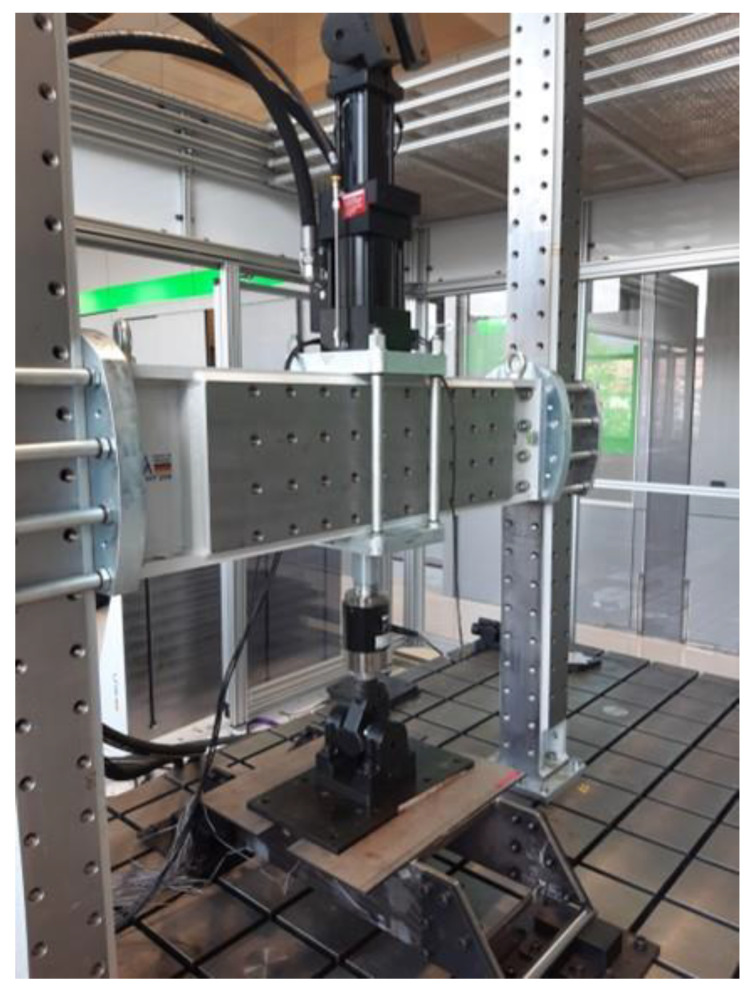
Use case and implemented scenario: scaled prototype of a bed of an industrial press installed in the fatigue test machine.

With the aim of replicating the working conditions of a real press, the prototype is tested using a large-scale fatigue-testing machine (see Figure 3). Compressive cyclic loading is applied at the top of the prototype, emulating the operating cycles of a real press. The amplitude of the cyclic loads has been calculated to be enough to cause fatigue damage at the critical points of the structure, with the objective of applying load cycles until the appearance of a fatigue crack. The details of the test are presented in Table 2.

The prototype is sensorized using a set of strain gauges. This set is divided between those gauges used as input sensors (used to provide real measurement data to feed the virtual sensing algorithms) and those gauges used as reference sensors (used only to check the performance of the VS results). During the test, strain at critical points of the prototype is estimated using VS algorithms. The number and location of installed sensors is detailed in Section 3.

Sensor data are collected using a data acquisition system. The software LabVIEW 2016 from National Instruments (NI), in combination with a rack NI CompactDAQ-9189 (with 8 slots), is used. An NI-9235 acquisition card is used for reading the strain gauges. All equipment is made by NI in Budapest, Hungary.

## 3. Theoretical Framework

This section is structured as eight subsections. In Section 3.1, the process followed for VS implementation is described. In Section 3.2, the system modelling is described. In Section 3.3, the model reduction is explained. In Section 3.4, the relation between displacements and strains is explained. In Section 3.5, Section 3.6 and Section 3.7, the different VS algorithms used are described. In the Section 3.8, the fatigue methods used are briefly explained.

### 3.1. Virtual Sensing Implementation

**Figure 4:**
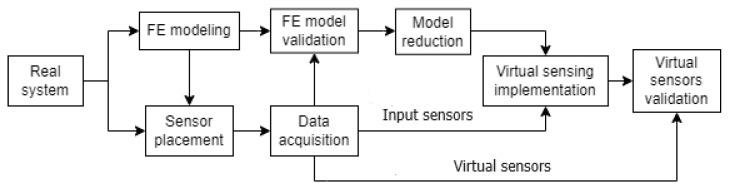
Flowchart of the process followed to implement and test VS algorithms.

The three selected VS algorithms are implemented in the use case defined in Section 2. The procedure followed for the VS implementation is described below (see Figure 4).

First, a structural FEM model of the system to be monitored is created. The model is used to identify the critical points of the system and to select the optimal placement for the real sensors. Then, a reduced model is extracted from the FEM model and, in combination with measurement data obtained from the real system, VS algorithms are implemented. Certain virtual sensors are created to estimate the strain at critical points of the prototype (operational virtual sensors), and other virtual sensors are installed at real sensorized points in the real system (REF virtual sensors) in order to validate the proper functioning of the VS algorithms.

### 3.2. System Modeling

To obtain a model capable of representing the behavior of a structure, an FEM model is generated from which mass, damping, and stiffness properties can be obtained. By applying the appropriate boundary conditions and external forces, it is possible to estimate different magnitudes at different points of the model, such as strain and stress. The 3D CAD model is generated using the software SolidWorks 2023, and the FEM model is generated and simulated using the software ANSYS 2023R2.

To calculate the evolution over time of an FEM model given defined external loads, the MCK (mass–spring–damping) equation is used (1). **M**, **C_D_** and **K** are stiffness, damping, and mass matrices, with the dimensions *n* × *n* (*n* being the number of degrees of freedom (DoFs) of the model). **q** is the displacement vector (with *n* × 1 dimensions). **f** is the vector of external forces (with the same dimensions as **q**). A more detailed process of how to obtain a state-space model from the matrices obtained from an FEM model can be seen in [26].

(1) $$M\ddot{q}\left(t\right)+C_{D}\dot{q}\left(t\right)+Kq\left(t\right)=f(t)$$

In a real industrial press, both static/quasi-static and dynamic loads can occur. In the experiment developed in this article, the only excitations present in the prototype are the cyclic loads applied by the fatigue machine in a controlled environment (quasi-static loads).

### 3.3. Model Reduction

Usually, FEM models of machines and industrial structures are highly complex, containing hundreds of thousands or millions of DoFs. This implies that, to perform simulations, excessive computing time is required. Lighter models are sometimes necessary to provide results in less time, for example, in real-time applications.

Model reduction methods allow for the simplification of full-size FEM models into simpler models (with a much smaller number of DoFs). Reduced models are able to replicate the behavior of the complete model (within a defined range), requiring much less computational power [27]. This result is of special interest in the field of VS, where it is important to be able to obtain estimations using the least possible processing capacity, especially when working in real-time processes is required.

In the case of static structural FEM models, assuming a model with a linear elastic behavior, all displacements of the model are related by the stiffness matrix **K**. There is also a proportional relationship between displacements and strains and between external loads and displacements [28].

One option for model reduction is to use Guyan’s Static Condensation [29]. This method condenses the complete model into a few selected DoFs (called “master DoFs”) providing reduced mass and stiffness matrices. To obtain a Guyan-reduced model for representing only the static and quasi-static behavior of a system, only the reduced stiffness matrix (**K**) is necessary.

Another option for model reduction is to describe the model using a limited number of modes, a method known as modal truncation [30,31]. The linear combination of a set of selected modes allows one to describe the state of deformation of the system at each instant of time, if the number and type of chosen modes are appropriate. In the resultant reduced model, each selected mode is a DoF.

If the selected modes are static modes (generally corresponding to the operational deflection shapes (ODS) of the system), a static modal truncation is performed. The resultant reduced model is able to describe the static and quasi-static behavior of the system, as long as all possible load cases are represented by the selected ODSs.

If the selected modes are normal modes (corresponding to the eigenvectors of the system, and their natural frequencies corresponding to the eigenvalues of the system), a modal truncation is performed. The resultant reduced model is able to describe the static, quasi-static, and dynamic behavior of the system, as long as the frequencies of external loads are within the frequency range of the selected modes.

In this article, Guyan’s Static Condensation is used in the case of the DSO algorithm because this reduction method allows one to obtain a reduced **K** matrix while maintaining the same Cartesian base as the original model. For the cases of the LSSE and the SSKF, the static modal reduction is used because this reduction allows one to represent (using very few DoFs) the different shapes in which the model can deform given a specific static or quasi-static load case. A normal modal truncation would be more appropriate if dynamic effects were expected in the model.

### 3.4. Strain–Displacement Relation

A strain matrix **G** can be obtained from an FEM model. This matrix relates the displacements of the model by the strain at specified points of the system (2). **G** has the dimensions *n* × *g*, *n* being the number of DoFs (**x**) of the reduced mode and *g* being the number of strains (collected in vector **g,** which has the dimensions 1 × *g*).

(2) g(t)=Gx(t)

To define **G**, the FEM model of the system must be used. For each DoF of the reduced model (1 to *n*), the strain results offered by the FEM model at each specified point are collected (ε_1,1_ to ε*_n_*_,*g*_) (3). If a Guyan-reduced model is used, each row of **G** is obtained by applying a unitary load at each DoF in a static analysis, keeping the rest of them fixed. If a static modal reduction is used, each row of **G** is obtained by applying reference forces in a static analysis in order to generate each ODS of the system. If a normal modal reduction is used, a normalized modal analysis must be performed with the FEM model. Each row of **G** is obtained by collecting the strains at the specified points for each mode shape.

(3) $$G=\left[\begin{array}{ccc} \varepsilon_{1,1} & \cdots & \varepsilon_{1,g}\\ \vdots & \ddots & \vdots \\ \varepsilon_{n,1} & \cdots & \varepsilon_{n,g} \end{array}\right]$$

### 3.5. Direct Strain Observer

If external force measurements are available and only static and quasi-static loads are expected, it is possible to use the stiffness matrix obtained from an FEM model to obtain the displacements for each time step of a simulation (4). Using the strain matrix **G**, it is possible to obtain the strain at specified points from the obtained displacements using (2).

(4) $$Kq\left(t\right)=f(t)$$

The DSO is an open-loop VS method, so the precision of the estimated measurements is limited by the precision with which the used model is able to replicate the behavior of the real structural system and by the quality of the input measurements. Since it is a deterministic method and there is no feedback data from the real system, there is no possibility of improvement of the estimations provided by the virtual sensors.

In the implementation of the DSO in this work, **K** is obtained using Guyan’s Static Condensation.

### 3.6. Least-Squares Strain Estimation

Least-Squares Strain Estimation (LSSE) is a deterministic model-based virtual sensing algorithm that allows one to estimate strain at unmeasured points of a structure from only strain data. This algorithm uses the strain matrix **G** and the Moore–Penrose pseudoinverse [32]. In the LSSE implementation, two sets of strain measurements must be defined: the measured strains vector **z_i_** (with the dimensions 1 × *i* dimension, *i* being the number of measured strains) and the virtual strains vector **z_v_** (with the dimensions 1 × *v*, *v* being the number of unknown strains).

In the LSSE implementation, the strain matrix **G** is divided in two parts: **G_i_** is corresponds to the measured strains (**z*_i_***) and has the dimensions *n* × *i* (5), while **G_vs_** is corresponds to the virtual strains (**z_v_**) and has the dimensions *n* × *v* (6).

(5) $$z_{i}\left(t\right)=G_{i}q(t)$$

(6) $$z_{vs}\left(t\right)=G_{vs}q(t)$$

Using generalized inverse **G_i_^+^**, (5) and (6) are combined in (7), allowing the estimation of unknown strains from a limited number of strain measurements.

(7) $$z_{vs}\left(t\right)=G_{vs}\left[{G_{i}}^{+}z_{i}\left(t\right)\right]$$

Compared to DSO, LSSE offers several advantages. It allows one to obtain strain estimates at unmeasured points even if force measurements are not available, using instead real strain gauges data. Also, LSSE allows for the use of a modal truncated model with several modes (as many as **G** includes), thus enabling one to work with different load cases. In the implementation of LSSE in this work, the static modal truncation is used.

When the number of input measurements *i* is equal to the number of virtual measurements *v*, statement (6) is determined, and a unique solution is found. If *i* is higher than *v*, then (6) is overdetermined, and if *i* is lower than *v*, then (6) is underdetermined. In both cases, the algorithm can provide a best-fit approximation of the solution.

So that LSSE can provide a good approximation of the solution (and thus obtain good virtual measurements), the condition number of matrix **G_i_** has to be low (close to 1). If the condition of **G_i_** is not low, then it is considered ill-conditioned, and significant errors can be expected in the output estimations of the algorithm.

### 3.7. Static Strain Kalman Filter

The Kalman Filter (KF) is a recursive Bayesian algorithm that estimates the hidden states of a system using a state-space model of the system and a limited number of measurements. This algorithm was first proposed by R. Kalman in 1960 [8], and since then, it has been used in a wide variety of fields, including the application of VS in machines and industrial structures [33,34].

The Static Strain Kalman Filter (SSKF) is a specific case of KF implementation used to estimate strain using a static model. It is a VS stochastic model-based algorithm which allows one to estimate the strain at unmeasured points from input strain measurements.

The state vector **x** contains as many states as there are DoFs considered in the model. In the implementation of the SSKF in this work, the static modal truncation is used, so each DoF corresponds to a static mode of deformation of the structure. The size of **x** is *1* × *n*, *n* being the number of states of the KF (corresponding to the number of static modes considered).

The model matrix **A** is created using an identity matrix with the dimensions *n* × *n* (8). **A** is discretized using (9) with Δt being the used time step.

(8) $$A=I_{n}$$

(9) $$A_{d}=e^{A∆t}$$

The measurement matrix **H** relates the measured strains with their corresponding states and has the dimensions *i* × *n*, *i* being the number of input strain sensors (10). **H** corresponds to the **G_i_** matrix, the part of **G** that contains the strain measurement points corresponding to the input gauges, as explained in (2). As with LSSE, SSKF allows one to work considering several static modes (as many as **G** includes) and is able to consider different load cases.

(10) $$H=G_{i}$$

Kalman filters manage the uncertainties associated with the model and the measurements using covariance matrices. **Q** is the covariance matrix associated with the model, and **R** is the covariance matrix associated with the measurements. If it is assumed that the states and the measurements are not correlated with each other, **Q** (11) and **R** (12) can be expressed as diagonal matrices, where the values of the diagonal correspond to the uncertainties associated with the states (q) and the measurements (i). The dimensions of these matrices are 2*n* × 2*n* and *i* × *i*, respectively.

(11) $$Q=diag(q_{1},q_{2}\ldots ,q_{2n})$$

(12) $$R=diag(r_{1},r_{2}\ldots ,r_{r})$$

SSKF is implemented in five substeps which are executed iteratively at each time step. In the first substep (13), a prediction of the present states (**x**(t)) is obtained using the model (**A**) and the corrected prediction of the previous iteration (**x**(t − 1)).

(13) $$x(t)=A_{d}x(t-1)$$

In the second substep (14), a prediction of the filter covariance (**P**(t)) is made using the corrected covariance of the previous iteration (**P**(t − 1)) and the covariance matrix of the model (**Q**).

(14) $$P(t)=A_{d}P(t-1){A_{d}}^{T}+Q$$

In the third substep (15), the gain of the filter is updated using the prediction of the filter covariance (**P**(t)), the measurement matrix (**H**), and the covariance matrix of the measurements (**R**).

(15) $$K(t)=P(t)H^{T}{\left(HP(t)H^{T}+R\right)}^{-1}$$

In the fourth substep (16), the prediction of the states is updated (**x**(t)**^updated^**) using the error between the measurements (**z**(t) − **Hx**(t)) and the calculated gain (**K**(t)).

(16) $${x(t)}^{updated}=x(t)+K(t)(z(t)-Hx(t))$$

In the fifth substep (17), the prediction of the filter covariance is updated (**P**(t)**^updated^**) using the calculated gain (**K**(t)).

(17) $${P(t)}^{updated}=P(t)-K(t)HP(t)$$

After each iteration of the SSKF, the virtual strain sensors’ values, **y**, are obtained from the states vector, **x**, through the outputs matrix, **C** (18). If it is of interest to obtain all the strain values (inputs and outputs) as output values of the filter, **C** corresponds to **G_st_** (with dimensions *n* × *g*), and **y** has the dimensions 1 × *g*.

(18) y(t)=Cx(t)

The observability is the capacity of the algorithm to obtain enough information from the real system to be able to estimate all the states of the used model. To determine if a KF is observable, an observability matrix (**O**) can be defined (19). If the rank of **O** is twice the number of states of the model (2*n*), then the KF is fully observable.

(19) $$O=\left[\begin{array}{c} \begin{array}{c} {A_{d}}^{T}H^{0}\\ {A_{d}}^{T}H^{1} \end{array}\\ \begin{array}{c} \vdots \\ {A_{d}}^{T}H^{2n-1} \end{array} \end{array}\right]$$

### 3.8. Fatigue Methods

Material fatigue is a phenomenon due to which a material can suffer damage because of repeated cyclic loads even though those loads are inferior to the ultimate strength of the material. The fatigue is a process that consists of two main phases: crack initiation and crack propagation. In the initiation phase, cyclic loads cause small deformations inside the material, generating stress concentration in certain areas, which causes the development of microcracks. In the propagation phase, the generated microcracks gradually extend into the material due to the cyclic loading. When a crack reaches a critical size, material failure occurs. Problems associated with material fatigue have a significant economic and safety impact in many engineering sectors [23].

The phenomenon of fatigue was described for the first time in the early 19th century, at the dawn of the Industrial Revolution. In the 1860s, A. Wohler developed SN curves (also known as Wohler diagrams). SN curves are graphical representations that show the relationship between the number of load cycles (N) and the stress level (S) required to produce failure in a material under cyclic load conditions [35].

In many materials (mainly metals), a behavior change from fatigue can be observed around N = 10^4^ [36]. Above that number of cycles, the amplitude of the stress cycles is below yield strength stress σ_YS_, and only elastic deformations occur in the material. This type of fatigue is known as high-cycle fatigue (HCF). Below that number of cycles, the amplitude of the stress cycles is between the ultimate tensile strength σ_UTS_ and σ_YS_, and permanent plastic deformations occur in the material. This type of fatigue is known as low-cycle fatigue (LCF) [37].

In the first half of the 20th century, the engineers Palmgren and Miner independently proposed what is today known as the Palmgren–Miner rule of damage [38]. Herein, *k* is the number of stress cycles, *n_i_* is the number of cycles at stress level *i*, *N_i_* is the fatigue life expected for the stress level *i*, and D is the accumulated fatigue damage (20). When D reaches 1, it is considered that the material has reached its fatigue life [38].

(20) $$D=\sum_{i=1}^{k}\frac{n_{i}}{N_{i}}$$

Rainflow Cycle Counting is an algorithm used for analyzing the fatigue of materials subjected to variable cycling loads and was presented by Endo in 1969 [39]. The algorithm provides a histogram of the load amplitudes, allowing one to calculate accumulated fatigue damage under varying loads.

When accumulated fatigue in the proximity of welds is studied, some considerations must be taken into account. In this article, the recommendations offered by the International Institute of Welding [40] are followed. To measure the effects of load cycles on weld toes, the Hot Spot stress method is used. This method allows one, from strain measurements at specific distances from the welds, to estimate the strain in the weld toes (which are considered the most critical in terms of accumulated fatigue but which are usually not feasible to measure directly due to their geometric characteristics). For accumulated fatigue in welds, specific SN curves are used, known as Fatigue Assessment Curves (FAT). These curves vary depending on the geometry of the welded components and the type of welding used as well as correction factors related to the thickness and geometry of the welded joints and the stress ratio (R) of the applied loads. FAT curves represent a 95% probability of survival.

## 4. Results

This section is structured in six subsections. In Section 4.1, the generated FEM model of the use case is described. In Section 4.2, the expected uncertainties are described. In Section 4.3, the number and location of installed sensors is described. In Section 4.4, the methods used to evaluate the results are described. In Section 4.5, the obtained results are presented, and finally, in Section 4.6, the obtained results are discussed.

### 4.1. Model of the Prototype

**Figure 5:**
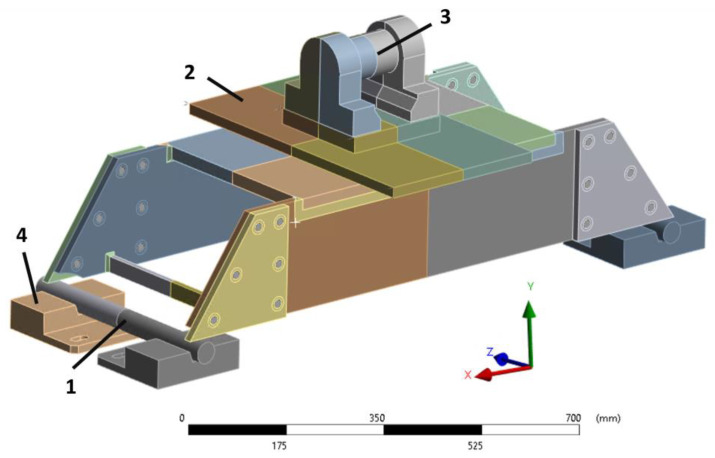
CAD model of the use case.

A detailed 3D CAD model of the scaled prototype was developed (see Figure 5). The model includes the external supports: two steel bars (see point 1 in Figure 5) each installed on two supports (see point 4 in Figure 5). The model also includes the steel plate (connected to the hydraulic piston of the fatigue machine) through which the loads are applied to the prototype (see point 2 in Figure 5).

From the CAD model, a FEM model is produced, defining the contacts between components, materials, and mesh properties. A dense mesh is used for the prototype in order to accurately estimate the strain, especially near the welded joints. External supports and the steel plate are also included in the FEM model with a lower meshing precision. The main specifications of the FEM model are shown in Table 3.

**Figure 6:**
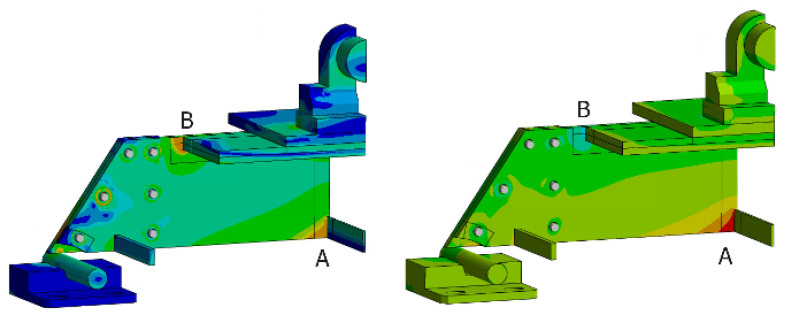
Distribution of equivalent Von Mises stress (**left**) and normal stress in X direction (**right**), in the FEM model. A cut view of the model is used for more clarity.

A static simulation is carried out with the FEM model (the results are shown in Figure 6) to identify the critical points of the prototype. Using first the equivalent von Mises stress, two critical points are identified (A and B). These points correspond to welded joints, so they are studied using the Hot Spot stress method. The comparison between the two points is detailed in Table 4. The formula to extrapolate the Hot Spot stress in a type A point (assuming a fine mesh) is shown in (21), with *t* being the thickness of the main plate [40].

The boundary conditions of the simulation are described below. Fixed supports are applied to the bottom of the bar supports (point 4 in Figure 5). The contacts between the bars (point 1 in Figure 5) and the supports are friction contacts. The contacts between the prototype and the bars and between the upper steel plate (point 2 in Figure 5) and the prototype are also friction contacts, all of them having a friction coefficient of 0.15. A centered 150 kN force is applied at point 3 in Figure 5. As neither the bars nor the prototype are fixed in the simulation, weak spring supports are added in order to avoid large displacements that could cause convergence issues in the calculation.

(21) $$\sigma_{HS\;type\;a}=1.67\sigma_{0.4t}-0.67\sigma_{1.0t}$$

**Figure 7:**
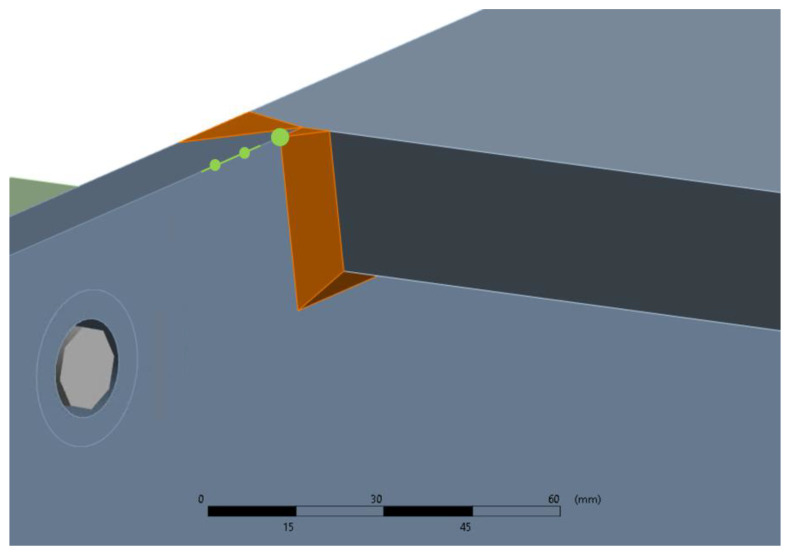
Detailed view of the welded joint and the exact location of point B (in green). The weld toe point is indicated with the big green dot. The stress extrapolation points at 0.4 t and 1.0 t are located 6 mm and 15 mm from the weld toe point, respectively (indicated with the small green dots).

**Figure 8:**
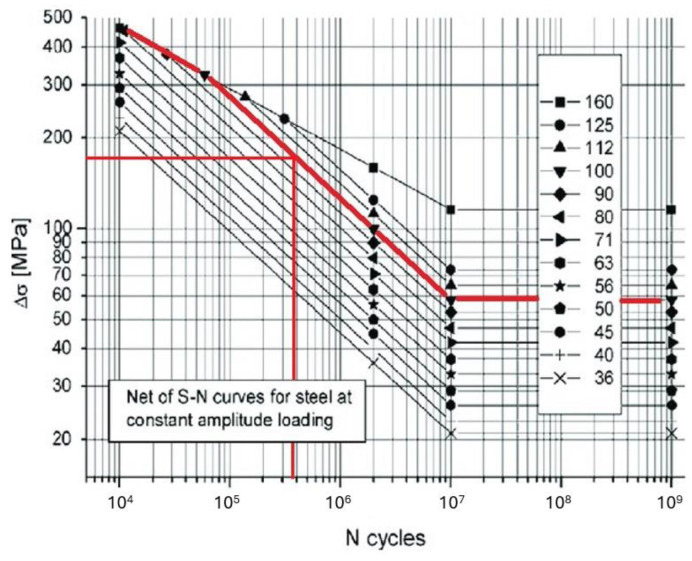
FAT SN curves for steel [40]. Marked in red is the FAT curve corresponding to the critical point B.

Point B is considered the most critical, and therefore, it is chosen as the point to be monitored using VS algorithms because it is the point at which a crack is expected to initiate during the experiment. Due to the biaxial symmetry of the prototype, there are four points B in total, which are all monitored simultaneously. A detailed view of point B is shown in Figure 7. The SN curve corresponding to point B is shown in Figure 8.

In the applied model reduction, three ODSs are used: one corresponding to the completely centered load application and two corresponding to the application of the load with a certain offset (in X in one and in Z in the other). The use of several operational deflection shapes allows the reduced model to better represent the strain differences between the four corners of the prototype. 

### 4.2. Expected Uncertainties

Different uncertainties are expected in the performed experiment. Geometric and manufacturing imperfections in the prototype, mainly related with the quality of the welds, may cause certain differences between the real prototype and the FEM model.

The placement of the prototype on its supports can also lead to a certain degree of uncertainty in the strain values of the real prototype because simple cylindrical supports without guides are used. The possible errors in the position and orientation of the strain gauges can also imply a certain degree of uncertainty in the measurements obtained.

The force value and the displacement of the piston are measured by sensors integrated into the fatigue-testing machine, but centering errors in force application can also be a source of uncertainty. Despite that, steel guides have been installed in the experiment in order to minimize decentering in the applied force.

### 4.3. Monitoring System

The location of the input sensors is chosen following two criteria: accessibility and ability to reach a significant deformation during the test. Based on these criteria, four strain gauges (gauges 1, 3, 4 and 5) are installed on the sides of the main frame, on the outer faces. These points are accessible in the case of a real machine in operation, and according to a static simulation performed with the FEM model, these points suffer from significant deformation. Monitoring these four points allows one to detect possible strain variations in each corner of the prototype.

**Figure 9:**
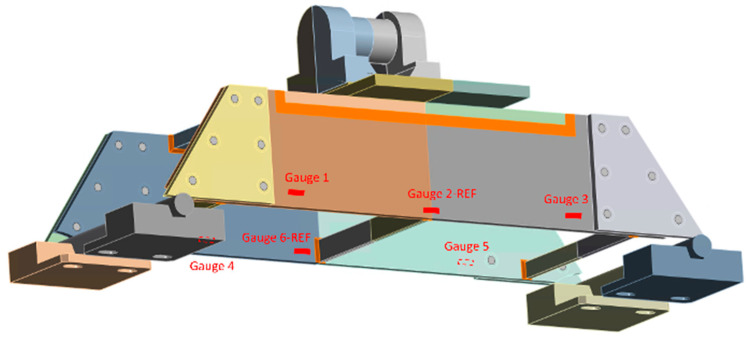
General view of the CAD model, with the locations of the gauges indicated.

For the virtual sensors, the following strategy is followed: First, virtual strain sensors are installed in the prototype at points where real gauges are installed. This is the case with gauge 2-REF and gauge 6-REF. Then, virtual strain sensors are located at points where real gauges are not installed (for reasons of unfeasibility). This is the case with the virtual sensors located at critical points B. Figure 9 shows graphically the position of the sensors in the prototype.

The six strain gauges are installed to measure strain in the X direction (see axis in Figure 5) because it is the main direction in which the prototype flexes under the applied load.

Since the four corners of the prototype are monitored using four input strain gauges, it is expected to not only estimate the number of cycles for crack initiation but also at which critical point of the four existing ones the crack will initiate. Each of these points is identified according to the number of the closest input gauge: B-1, B-3, B-4, or B-5.

In addition to the strain gauges, the fatigue test machine provides a measurement of the force applied at each moment. This measurement can be used as a sensor input for any of the implemented VS algorithms.

### 4.4. Evaluation Methods

A set of indicators is used to evaluate the strain estimations obtained in the REF virtual sensors and does so by comparing the virtual measurements with real measurements, with the aim of checking the correct functioning of the VS algorithms.

The main evaluation method used in this article is the Relative Root Mean Square Error (RRMSE) (22), with “ref” being the measured signal and “est” being the signal as estimated by a VS algorithm. RRMSE is used to evaluate the error in magnitude of the estimated signal with respect to the reference signal.

(22) $$RRMSE\left[\%\right]=\frac{RMSE(ref,est)}{mean(ref)}\times 100$$

Moreover, the relative error of the mean and of the range between the estimated signals and the reference signals is shown. In parallel, the accumulated damage in B-1, B-3, B-4, and B-5 is estimated using strain virtual sensors and the Hot Spot stress extrapolation, using the corresponding FAT SN curve at these critical points.

The number of cycles before crack initiation as well as at which of the four critical points the crack initiates will be compared to the observations in the experiment.

### 4.5. Results

**Figure 10:**
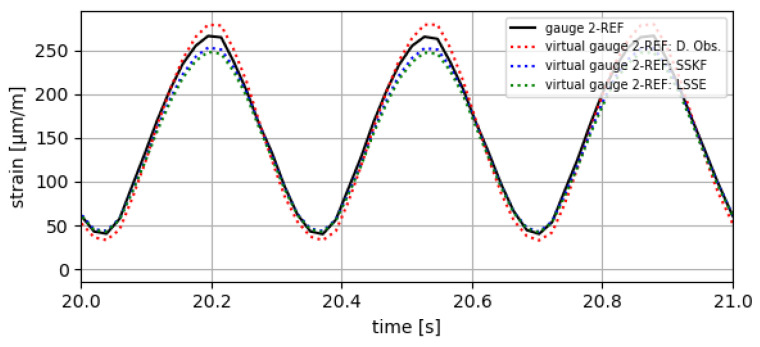
Results of virtual strain obtained at gauge 2-REF with the tested VS algorithms.

**Figure 11:**
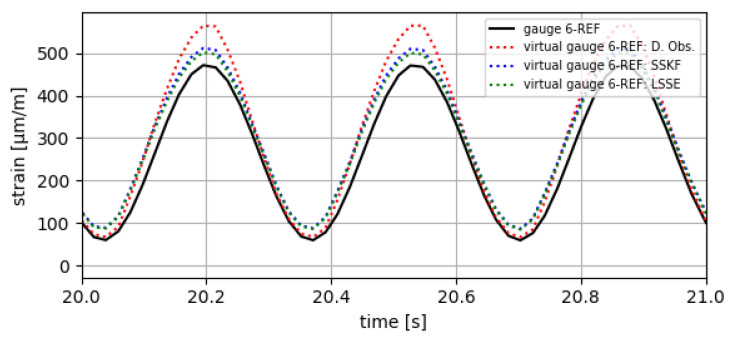
Results of virtual strain obtained at gauge 6-REF with the tested VS algorithms.

The results obtained are divided into two parts. In the first part, the estimates obtained at the points defined as reference (gauge 2-REF and gauge 6-REF) are compared with data measured in the prototype at those same points, with the aim of checking the correct functioning of the implemented VS algorithms. The results of this comparison are shown in Figure 10 and Figure 11 and in Table 5, Table 6 and Table 7.

In the second part, the implemented VS algorithms (LSSE and SSKF) are used to estimate the Hot Spot stress at the four critical points of the prototype (B-1, B-3, B-4, and B-5). With the results of these virtual sensors, the number of cycles required for crack initiation is estimated. The point (of the four possible mentioned) with the lowest number of cycles before crack initiation is the point at which the crack is expected to initiate. Results are summarized in Table 8.

**Figure 12:**
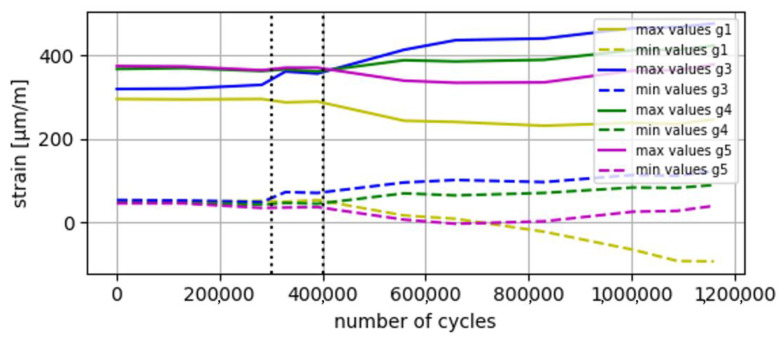
Evolution of the values of the input strain gauges during the experiment (max values and min values). A change in the behavior of the prototype (mainly captured by gauges 1 and 3) is observed between *n* = 300,000 and 400,000 (see interval between dashed lines), attributable to the beginning of a crack in B-1.

**Figure 13:**
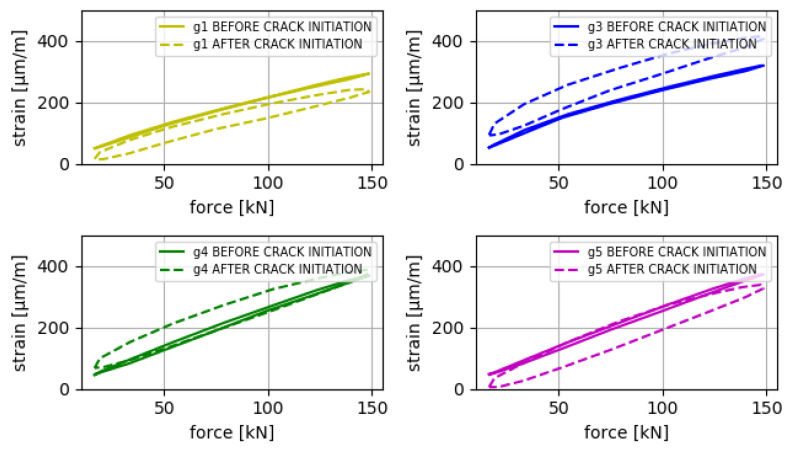
Relationship between measured strain and applied force in the prototype. The loading and unloading phases of a force cycle are observed. The continuous lines correspond to a load cycle before crack initiation (*n* = 130,000), and the dashed lines correspond to a load cycle after crack initiation (*n* = 550,000). It can be observed that, after the appearance of a crack, a significant hysteresis appears between the loading and unloading phases, which verifies a change in the behavior of the prototype.

**Figure 14:**
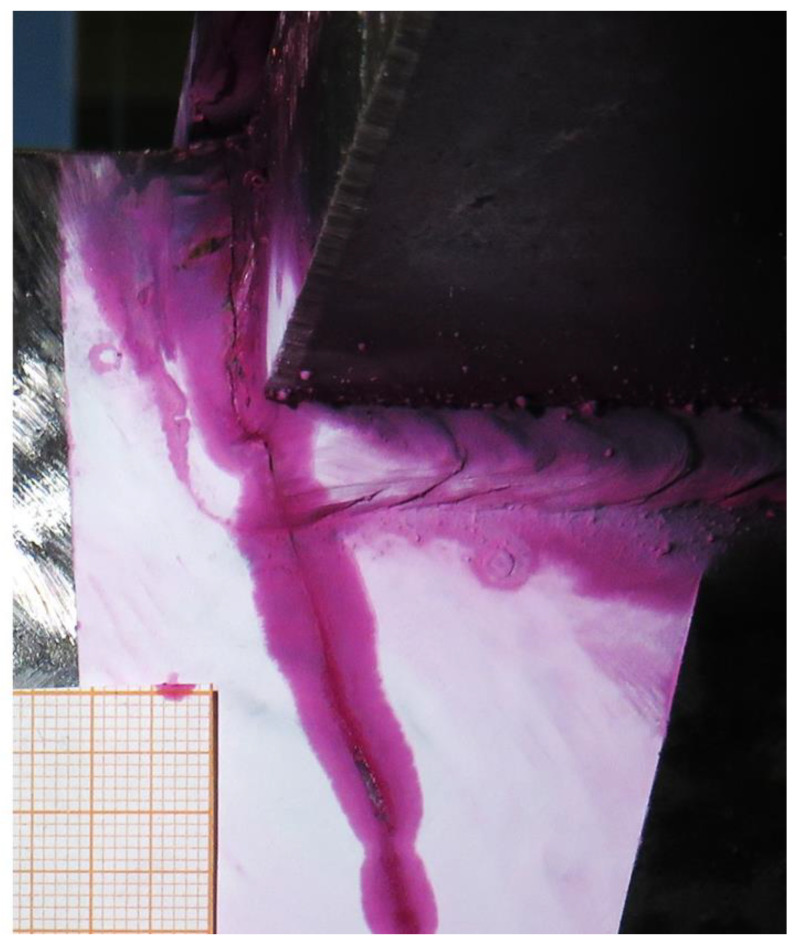
Crack generated in the weld of critical point B-1, highlighted with pink penetrating liquid. The experiment stopped at *n* = 1,150,000, with the crack reaching a significant size.

The results obtained through the estimates of the VS algorithm are validated with the results obtained in the real experiment, specifically the number of cycles before crack initiation and the critical point where the crack starts. The results of the validation are shown in Figure 12, Figure 13 and Figure 14.

### 4.6. Results Discussion

In the first phase of the VS validation, three different algorithms for strain estimation have been tested (DSO, LSSE, the SSKF), comparing the strain estimates obtained with the virtual sensors to the real measurements from the same points. It has been observed that DSO provides a certain degree of error in its estimates (with an RRMSE above 20% in some cases), while LSSE and SSKF significantly improve their results (with an RRMSE below 15% in all cases).

In the second phase of the VS validation, the accumulated damage at the identified critical points of the prototype is estimated, with the aim of estimating the number of cycles until crack initiation. The DSO only uses one static mode, so it is not able to differentiate between the four defined critical points, offering only a generic non-conservative result of 258,000 cycles until crack initiation for all four critical points. Both LSSE and SSK have indicated that the number of cycles until crack initiation is approximately 300,000 at the critical point B-1. The FEM model indicates that crack initiation will occur at 370,000 cycles, and experimental results show that the crack initiated between 300,000 and 400,000 cycles in B-1. Therefore, the results offered by these last two algorithms can be considered positively validated. On the contrary, DSO gives a result with certain degree of error.

Both LSSE and SSKF provide satisfactory VS results and also allow one to identify the break point (from a selected set of critical points) because they use several static modes to describe the system as well as more input measurements. Furthermore, in the case of SSKF, feedback data are used to correct the provided estimations. On the other hand, DSO shows a certain degree of error between the estimates provided and the experimental data, and it cannot identify the exact point of crack initiation. Due to this, both LSSE and SSKF are considered better options when implementing a VS system in real industrial systems as compared to the use case presented in this article.

Taking into account the results of both the first and second phases of validation, it can be stated that LSSE is the algorithm (of the algorithms tested in this work) that offers the best virtual strain results. In addition, it has the advantage over SSKF of being simpler to implement because it does not contain tuning parameters that the user must adjust (parameters that have a significant effect on the results obtained).

## 5. Conclusions

In this article, it has been demonstrated that is possible to monitor the strain and stress at unmeasured points of a structure from limited strain measurements using virtual sensing methods. It has also been demonstrated that, using the strain and stress estimated with VS algorithms, is possible to estimate with reasonable precision the number of cycles until the appearance of a crack in a structure subjected to cyclic loads.

Three different VS algorithms have been tested in this article. One of them, DSO, requires measurement of the external loads on the system. The other two, LSSE and SSKF, do not require measurement of the external loads on the system, using strain measurements as inputs instead. These last two algorithms also allow one to identify the most critical point in terms of fatigue (within a preselected set of critical points). Regarding the results obtained from the experiment carried out, SSKF and LSSE have shown greater accuracy than DSO in the estimated measurements. Between SSKF and LSSE, the accuracy obtained in the estimated measurements is very similar, but due to its greater simplicity of implementation, LSSE can be considered the most suitable VS algorithm of the three presented in this article.

The limitations of the results obtained in this experiment must be taken into account. The VS algorithms presented in this work can only be applied in systems subjected to static or quasi-static loads and are not applicable in cases where dynamic effects are present. Moreover, these VS algorithms can only work with time-invariant models.

The knowledge that can be drawn from the results of the experiment shown in this article may be useful in systems such as industrial presses and similar equipment, with the aim of being used to optimize maintenance operations in order to reduce costs and to help prevent potential failures due to fatigue before they occur.

The work presented in this article can be continued by following different research lines. An interesting line of work would be to continue the experiment presented in this article but focusing on the monitoring of the crack-growth phase. Another interesting line of work would be to focus on using VS algorithms for detecting behavior changes in order to perform model updating, which means updating the model parameters so that the model can reflect the evolution of the real system. This line of work would especially be of interest integrated into a more complex digital twin environment, for example, applied for crack detection and crack growth monitoring in industrial machines.

## Figures and Tables

**Table 1:** Main specifications of the bed press prototype.

Feature	Value
Weight	127 kg
Length	1120 mm
Height	210 mm
Width	410 mm
Main plates thickness	15 mm
Reinforcement bars thickness	10 mm
Material	Steel S-275 (made by ArcelorMittal in Olaberria, Spain)
Supports	4 simple supports

**Table 2:** Characteristics of the fatigue test performed.

Feature	Value
Type	Sinusoidal
Max load	150 kN
Min load	15 kN
Stress ratio (R)	0.1
Mean load	82.5 kN
Frequency	3 Hz
Type	Sinusoidal
Max load	150 kN

**Table 3:** Main specifications of the FEM model.

Feature	Value
Number of elements	2,140,000
Element order	Quadratic
Element size	Generally 5 mm, 2 mm near welded joints.

**Table 4:** Comparison between the criticality of points A and B using the Hot Spot stress method and the FAT.

Point	HS Type	HS Stress [MPa]	FAT	FAT Corrected	N° Cycles for Crack Initiation
**A**	**A**	167.8	100	110.8	575,000
**B**	**A**	174.5	90	99.7	370,000

**Table 5:** Virtual strain results obtained at gauge 2-REF and gauge 6-REF with the DSO algorithm.

Point	DSO		
	RRMSE [%]	e. Mean [%]	e. Range [%]
Gauge 2-REF	6.6	0.6	7.5
Gauge 6-REF	22.1	18.1	21.2

**Table 6:** Virtual strain results obtained at gauge 2-REF and gauge 6-REF with the SSKF algorithm.

Point	DSO		
	RRMSE [%]	e. Mean [%]	e. Range [%]
Gauge 2-REF	7.0	5.6	10.1
Gauge 6-REF	13.4	12.1	1.8

**Table 7:** Virtual strain results obtained at gauge 2-REF and gauge 6-REF with the LSSE algorithm.

Point	DSO		
	RRMSE [%]	e. Mean [%]	e. Range [%]
Gauge 2-REF	6.8	5.5	8.1
Gauge 6-REF	13.3	12.2	1.3

**Table 8:** Comparison of the estimated number of cycles necessary for crack initiation by VS algorithms.

Algorithm Used	Number of Cycles for Crack Initiation			
	B-1	B-3	B-4	B-5
DSO	258,000			
SSKF	301,090	332,273	495,913	486,181
LSSE	300,305	331,407	494,619	484,911

## Data Availability

The data presented in this study are available on request from the corresponding author. The data are not publicly available due to privacy restrictions.
